# Blood Leukocyte Counts in Alzheimer Disease

**DOI:** 10.1001/jamanetworkopen.2022.35648

**Published:** 2022-10-10

**Authors:** Jiao Luo, Jesper Qvist Thomassen, Børge G. Nordestgaard, Anne Tybjærg-Hansen, Ruth Frikke-Schmidt

**Affiliations:** 1 Department of Clinical Biochemistry, Copenhagen University Hospital–Rigshospitalet, Copenhagen, Denmark; 2 Department of Clinical Epidemiology, Leiden University Medical Center, Leiden, the Netherlands; 3 Section of Gerontology and Geriatrics, Department of Internal Medicine, Leiden University Medical Center, Leiden, the Netherlands; 4 Department of Clinical Biochemistry, Copenhagen University Hospital–Herlev and Gentofte, Herlev, Denmark; 5 The Copenhagen General Population Study, Copenhagen University Hospital–Herlev and Gentofte, Herlev, Denmark; 6 Department of Clinical Medicine, Faculty of Health and Medical Sciences, University of Copenhagen, Copenhagen, Denmark

## Abstract

**Importance:**

Emerging evidence implicates a role for neuroinflammation in Alzheimer disease (AD) pathogenesis, predominantly involving the innate immune system. Blood leukocyte counts are easily accessible markers of immune function; however, their association with the risk of AD is unknown.

**Objective:**

To investigate the observational and genetic associations between types of blood leukocytes and risk of AD.

**Design, Setting, and Participants:**

In a cohort study comprising observational and genetic analyses, the Copenhagen General Population Study prospective cohort (n = 101 582) was used for the observational analyses. For the genetic studies, nonlinearity was first evaluated for the association between leukocyte cell counts and AD risk using individual-level data from the UK Biobank (n = 365 913). Subsequently, a 2-sample mendelian randomization framework was applied using genetic instruments for blood leukocyte counts (n = 563 085); for AD, the European Alzheimer & Dementia Biobank was used, including 85 934 individuals with AD and 401 577 controls and the International Genomics of Alzheimer’s Project, including 21 982 individuals with AD and 41 944 controls.

**Exposures:**

Observational and genetically determined types of blood leukocyte counts.

**Main Outcomes and Measures:**

Hazard ratios (HRs) and 95% CIs for AD of cell count percentile groups in observational studies and odds ratios (ORs) and 95% CIs for AD per 1 SD genetically determined cell counts.

**Results:**

This cohort study included 101 582 participants (55 891 [55.0%] women) with a median age of 58 years (IQR, 48-67 years); of these, 1588 individuals developed AD. Multivariable-adjusted HRs for participants in the less than 5th vs the 25th to 75th (reference) percentile group were 1.24 (95% CI, 0.99-1.54) for blood monocytes and 1.25 for blood eosinophils (95% CI, 1.05-1.50). For participants in the greater than 95th vs the 25th to 75th percentile group, the HR was 1.30 (95% CI, 1.06-1.61) for blood neutrophils. Genetically, no evidence favored possible nonlinear associations. The ORs for AD per 1-SD decrease in genetically determined blood monocytes were 1.04 (95% CI, 1.00-1.10) in the European Alzheimer & Dementia Biobank consortium and 1.09 (95% CI, 1.01-1.17) in the International Genomics of Alzheimer’s Project consortium. Using mendelian randomization, sensitivity analyses and multivariable analysis showed similar results.

**Conclusions and Relevance:**

The findings of this study suggest that low blood monocyte counts are associated with increased AD risk. These findings highlight a potential role of the innate immune system in AD pathogenesis.

## Key Points

**Question:**

What is the relevance of types of blood leukocytes in Alzheimer disease (AD)?

**Findings:**

In this cohort study of 101 582 individuals in the Danish general population, low baseline blood monocyte counts were associated with increased AD risk. In a mendelian randomization framework using the most powerful genomic data sets, genetically determined low monocyte counts were also associated with increased AD risk, an association independent of other types of blood leukocytes.

**Meaning:**

The findings of this study suggest that the observational and genetic association observed between low monocyte counts and increased risk of AD highlights a possible role of the innate immune system in AD pathogenesis.

## Teaser

This cohort study examines the association between blood leukocyte counts and Alzheimer disease pathogenesis, using observational and genetic analysis.

## Introduction

Worldwide, Alzheimer disease (AD) is the most prevalent cause of dementia in the older population, and the number of affected individuals is increasing due to aging populations.^1^ Emerging evidence implicates a role for neuroinflammation in AD pathogenesis and progression, predominantly involving the innate immune system.^2^ Types of blood leukocytes are important and easily accessible markers of immune function. However, the association between blood leukocyte counts and AD remains unknown.

Several genetic variants associated with AD have been discovered in genome-wide association studies (GWAS) and are mapped to genes that are closely involved in regulating the innate immune function both within the central nervous system and in the blood, for example, genes that encode proteins involved in regulation of complement activation and phagocytic function of myeloid cells.^3,4,5,6,7^ The recent landmark study of AD genomics also highlighted gene sets related to immunity, including macrophage and microglial activation.^8^ Furthermore, experimental studies have shed light on the possible mechanisms of blood-derived macrophages and monocytes in AD. Particularly, circulating monocytes are reported to engulf and remove amyloid-β (Aβ) peptide, a major pathologic hallmark of AD, in the peripheral bloodstream.^9^ This action subsequently facilitates the clearance of brain Aβ via a physiologic equilibrium between the periphery and brain.^10^ Moreover, during neuroinflammation involving blood-brain barrier leakage, monocytes can migrate into the brain upon recruitment by chemoattractants and differentiate into microglialike macrophages for Aβ removal.^11^ Other studies also found that infiltrating neutrophils upon neuroinflammation crosstalk with the immune system in the brain and may produce neurotoxic molecules and induce hypoperfusion by adhering to brain capillaries and reducing cortical blood flow.^12,13^ Other blood leukocyte types may also play a role in AD pathogenesis; however, their mechanisms are less well studied.

Few epidemiologic studies, mostly with small-scale case-control designs, have explored the associations between types of blood leukocyte counts and risk of AD, and the results are conflicting.^14,15,16^ It thus remains unclear whether changes in types of blood leukocyte counts, as a reflection of immune dysregulation, contribute to AD risk.

Therefore, we investigated the associations between types of blood leukocytes (lymphocytes, monocytes, neutrophils, basophils, and eosinophils) and AD. We first performed a prospective cohort analysis in the Copenhagen General Population Study (CGPS) including 101 582 individuals. Second, to examine potential involvement in pathogenic pathways, we performed nonlinear mendelian randomization (MR) among 365 913 participants in the UK Biobank and a 2-sample MR study including more than 1 million participants to investigate the association of genetically determined types of blood leukocytes with risk of AD.

## Methods

Our study followed the Strengthening the Reporting of Observational Studies in Epidemiology (STROBE) reporting guideline using cohort studies and MR design.

### Observational Analyses

#### Study Population

We included participants from the Copenhagen General Population Study (CGPS) (eMethods in Supplement 1). The study was approved by institutional review boards and Danish ethical committees and was conducted according to the Declaration of Helsinki.^17^ Written informed consent was obtained from all participants. We used data from 101 582 participants without any prevalent dementia at baseline in the present study.

Incident AD events were documented from the national Danish Patient Registry (eMethods in Supplement 1) and defined according to code 290.10 in the *International Classification of Diseases, Eighth Revision* and codes F00 and G30 in *International Classification of Diseases, Tenth Revision*.

#### Observational Statistics

Missing values (eTable 1 and eTable 2 in Supplement 1) were imputed using multiple imputation by chain equations. We used Cox proportional hazards regression models for analyses based on cell counts categories and on a linear scale of cell counts using restricted cubic splines. An in-depth description of the statistical analyses and sensitivity analyses is provided in the eMethods in Supplement 1.

### Genetic Analyses

We performed MR analyses to assess the association between genetically determined cell counts with risk of AD. A schematic overview and an explanation of the MR design are given in eFigure 1 and the eMethods in Supplement 1. The UK Biobank data analyses were performed under the No. 66214 project approval.

Summary statistics of leukocyte counts were retrieved from the largest published GWAS on blood cell traits conducted in 563 085 participants of European ancestry from 26 cohorts (eMethods in Supplement 1).^18^ Independent single nucleotide variants (SNVs) at a genome-wide significance level (*P* < 5 × 10^−8^) were selected as genetic instrumental variables. The final βcoefficient was per 1-SD increase in inverse-rank normalized log_10_-transformed leukocyte counts per additional effect allele.

#### Nonlinear MR

Nonlinear MR analyses were performed to assess potential nonlinear associations between cell counts and AD, using individual-level data from 365 913 nonrelated White British participants in the UK Biobank. This approach assessed how the associations of cell counts with AD differ across various groups of instrument-free cell counts (residuals of cell counts regressing on genetic instrumental variables). Piecewise linear MR estimates within each stratum were generated, referred to as localized average causal effects. Two statistical nonlinear tests are presented: Cochran *Q* statistic assesses differences in MR estimates across different groups and quadratic test for the trend between exposure and localized average causal effects values. A detailed description is available in the eMethods in Supplement 1.

#### Two-Sample MR

Genetic associations for late-onset AD were obtained from the largest consortium—the European Alzheimer & Dementia Biobank (EADB)—which brings together a range of European GWAS consortia, and summary estimates were based on 39 106 individuals with clinically diagnosed AD, 46 828 proxy-AD cases (from the UK Biobank), and 401 577 controls.^8^ To rule out the possible influence of proxy AD cases, we additionally used data from the International Genomics of Alzheimer’s Project (IGAP), which meta-analyzed GWA studies on individuals of European ancestry comprising 21 982 individuals with an AD diagnosis or autopsy-confirmed AD and 41 944 cognitively healthy individuals as controls from 4 consortia.^19^ Detailed descriptions are in the eMethods in Supplement 1.

We performed primary univariable MR analyses using inverse-variance weighted (IVW), and sensitivity analyses including weighted median estimator, MR-Egger regression, and MR-PRESSO^20,21,22^ (eMethods in Supplement 1). The MR results are expressed as odds ratios (ORs) with corresponding 95% CIs on AD risk per 1-SD increase in genetically determined types of leukocytes. We generated scatterplots to visualize the genetic estimates from different MR methods. Pleiotropy was tested by the asymmetry in funnel plots, displaying the genetic estimates on the horizontal axis vs their square root precision on the vertical axis. Furthermore, we calculated the statistical power for MR analyses using online power calculation.^23^

We additionally performed multivariable MR (MVMR) for all leukocyte types to account for potential unbalanced horizontal pleiotropy. The MVMR estimates the effects of multiple related exposures on an outcome and the direct genetic effect of each exposure is obtained taking mediation by other traits into account.^24^ Genetic variants associated with any of the types of leukocytes were considered as instrumental variables (eTables 1-6 in Supplement 2).

### Statistical Analysis

All statistical analyses and figures were generated using R statistical software, version 4.1.0 (R Foundation for Statistical Analysis). A 2-sided *P* value <.05 was considered as statistically significant.

## Results

### Observational Analyses

We included 101 582 individuals (55 891 [55.0%] women, 45 691 [45.0%] men) from the Copenhagen General Population Study who were followed up for 944 381 person-years. Median age was 58 (IQR, 48-67) years. A total of 1588 individuals were diagnosed with AD during a median follow-up time of 9.4 (IQR, 6.7-11.9) years. Baseline characteristics of the participants are displayed in eTable 3 in Supplement 1.

**Figure 1.**
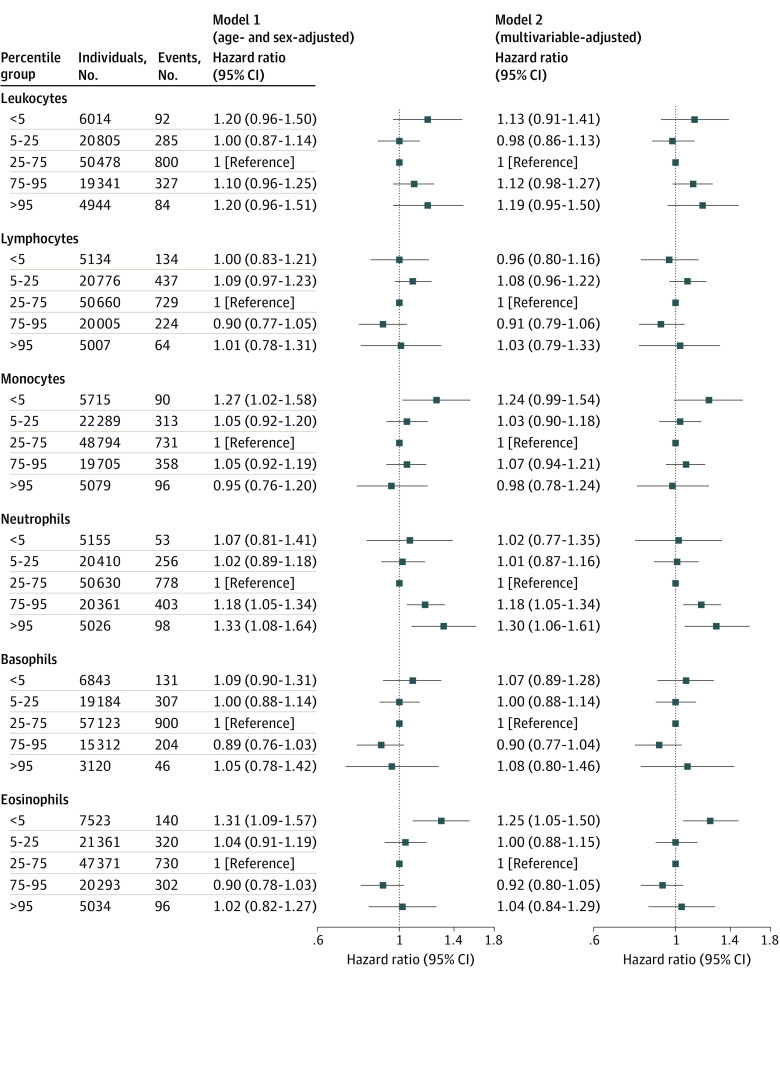
Types of Blood Leukocyte Counts in Percentile Categories and Risk of Alzheimer Disease in the Copenhagen General Population Study Cox regression model 1 was adjusted for age (time scale) and sex; model 2 was additionally adjusted for educational levels, *APOE* ε2/3/4 genotype, and lifestyle factors including body mass index, smoking status, alcohol consumption, physical activity, hypertension, and type 2 diabetes. Hazard ratios for percentile groups are compared with the 25th-75th percentile group (reference).

In the age- and sex-adjusted Cox proportional hazard model (model 1), hazard ratios (HRs) for individuals in the less than 5th vs the 25th to 75th percentile group (corresponding absolute levels presented in eTable 4 in Supplement 1) were 1.27 (95% CI, 1.02-1.58) for blood monocytes and 1.31 (95% CI, 1.09-1.57) for blood eosinophils (Figure 1). Hazard ratios for individuals in the 75th to 95th and greater than 95th vs the 25th to 75th percentile group were 1.18 (95% CI, 1.05-1.34) and 1.33 (95% CI, 1.08-1.64) for blood neutrophils. In the multivariable-adjusted model (model 2) including educational level and *APOE* genotype, these associations were slightly attenuated (Figure 1). Furthermore, in a complete case sensitivity analysis results were similar (eFigure 2 in Supplement 1).

**Figure 2.**
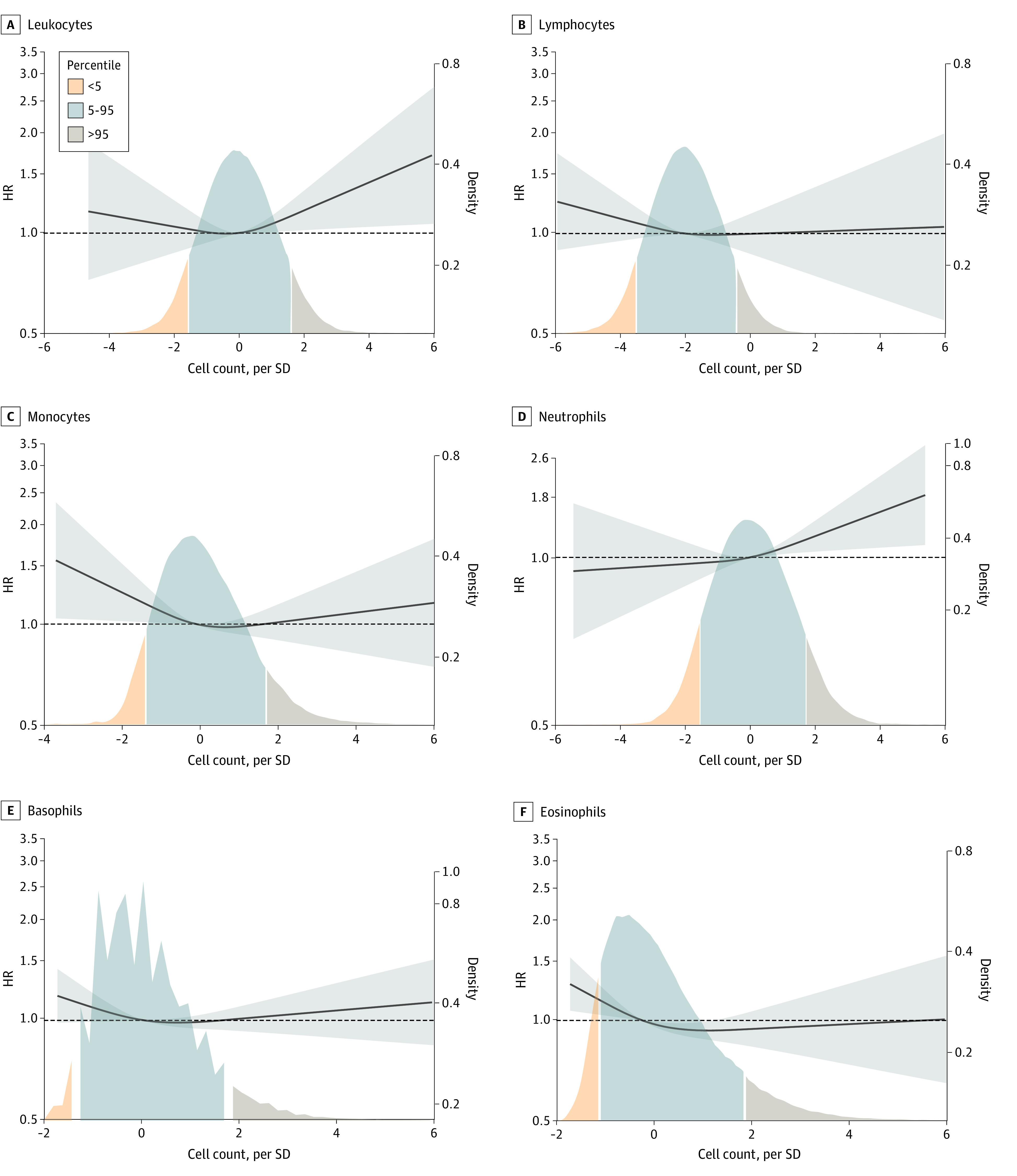
Hazard Ratios for Alzheimer Disease as a Function of Types of Blood Leukocyte Counts on a Continuous Scale in the Copenhagen General Population Study Results were derived from Cox regression models adjusted for age (time scale) and sex, educational levels, *APOE* ε2/3/4 genotype, and lifestyle factors including body mass index, smoking status, alcohol consumption, physical activity, hypertension, and type 2 diabetes. Solid lines represent hazard ratios and gray shadowed areas represent corresponding 95% CIs, using restricted cubic splines with knots at the 5th, 50th, and 95th percentiles and with median leukocyte counts as reference. The density plots indicate the distribution of the population.

Restricted cubic spline analyses examined the risk of AD by types of leukocytes in multivariable-adjusted regression models (Figure 2). Low counts of blood monocytes and eosinophils and high counts of blood leukocytes and neutrophils were associated with higher risk of AD compared with the corresponding median counts. Nonlinearity was assessed by comparing 2 models with and without nonlinear terms using likelihood ratio tests, with *P* = .12 for leukocytes, *P* = .38 for lymphocytes, *P* = .07 for monocytes, *P* = .35 for neutrophils, *P* = .07 for basophils, and *P* = .08 for eosinophils.

### Genetic Analyses

Independent genetic SNVs were used as instrumental variables separately for leukocytes (n = 259), lymphocytes (n = 394), monocytes (n = 380), neutrophils (n = 238), basophils (n = 350), and eosinophils (n = 135). These SNVs explained the variation in the blood leukocyte counts by 3.4%; lymphocytes, 3.4%; monocytes, 9.7%; neutrophils, 7.5%; basophils, 8.8%; and eosinophils, 2.6%. Summary information is given in eTable 5 in Supplement 1 and detailed descriptions of the instrumental variables are given in eTables 1-6-in Supplement 2.

#### Nonlinear MR

**Figure 3.**
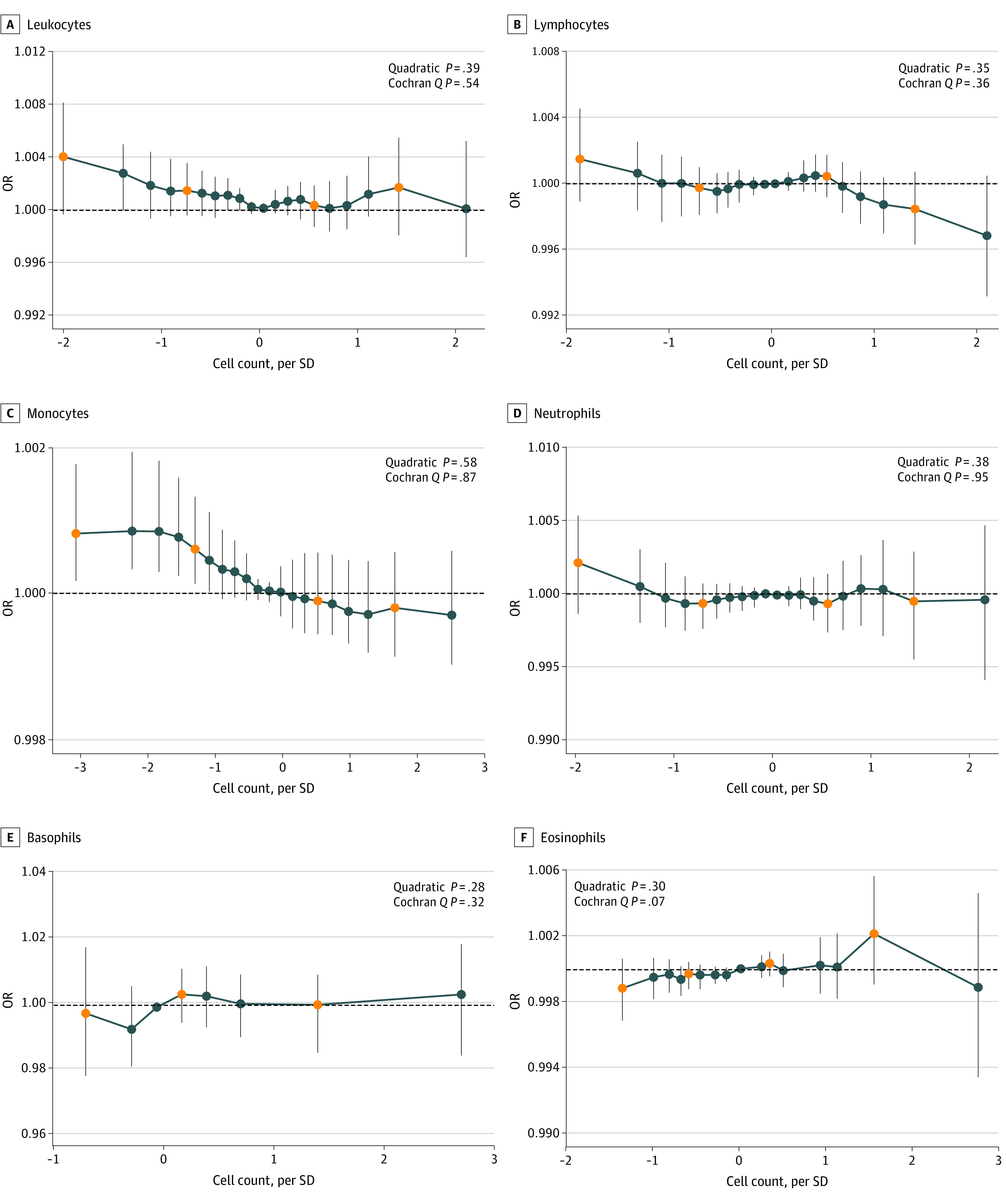
Nonlinear Mendelian Randomization of Types of Blood Leukocyte Counts on Risk of Alzheimer Disease in the UK Biobank Plots were generated using the piecewise linear model from nonlinear mendelian randomization analysis. The model builds 20 equally sized groups according to the residual variation of cell counts. Dots with vertical lines represent the localized average causal effects (95% CIs) in each stratum, in alignment with the observational analyses when applicable. Groups for basophil and eosinophil counts are less than 20 owing to a lower number of possible counts and thus the same values for some percentiles; for basophils, the 3 orange dots represent the 30th, 75th, and 95th percentile groups. For eosinophils, the 4 orange dots represent the 5th, 25th, 75th, and 95th percentile groups. The *P* values presented for genetic nonlinear relationships are from the piecewise linear model (quadratic and Cochran *Q*).

Nonlinear MR analyses were performed with a piecewise linear method using 20 strata (per 5 percentiles where available) according to the residual variation of cell counts. The results indicated linear associations between genetically predicted types of leukocytes and AD (*P* values for the Cochran *Q* test ranged from *P* = .07 for eosinophils to *P* = .95 for neutrophils, and *P* values for the quadratic test ranged from *P* = .28 for basophils to *P* = .58 for monocytes), suggesting no evidence for nonlinear genetic associations between cell counts and AD (Figure 3). The localized average causal effects estimates also revealed associations between low monocyte counts and modestly increased risk of AD.

#### Two-Sample MR

We had 80% power with α = .05 to detect a causal OR for AD ranging from 1.08 or higher for monocytes to 1.15 or higher for basophils in both EADB and IGAP (eFigure 3 in Supplement 1).

**Figure 4.**
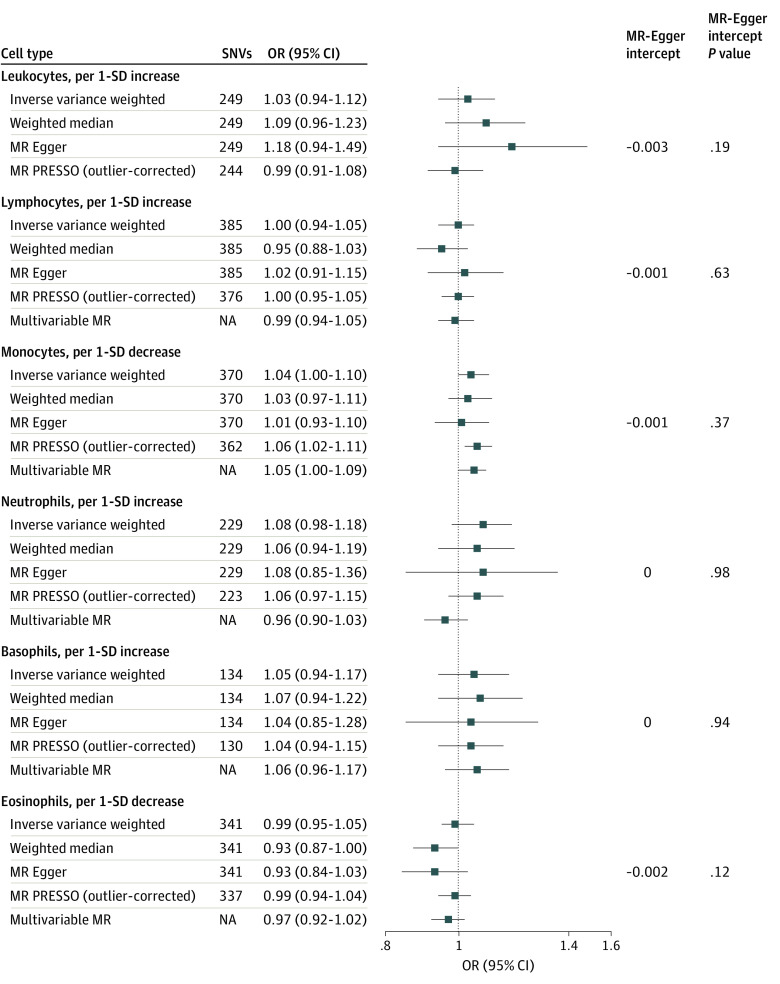
Mendelian Randomization (MR) Estimates of Types of Blood Leukocyte Counts on Risk of Alzheimer Disease Using the European Alzheimer & Dementia Biobank Estimated odds ratios (ORs) represent the effect of per 1-SD increase or decrease in different leukocyte counts on risk of Alzheimer disease obtained from different methods. *P* values for MR-Egger intercept represent horizontal pleiotropy. Total leukocyte count was not in the multivariable MR because the counts of subpopulations were derived from the total leukocyte count. NA indicates not applicable; SNVs, single nucleotide variants.

Mendelian randomization results from the EADB are presented in Figure 4. Odds ratios for AD per 1-SD decrease of genetically determined blood monocyte counts were 1.04 (95% CI, 1.00-1.10) in the primary IVW analysis, 1.06 (95% CI, 1.02-1.11) in the outlier-corrected MR PRESSO analysis, and 1.05 (95% CI, 1.00-1.09) in the MVMR analysis. No associations with AD were observed for blood lymphocytes, neutrophils, basophils, and eosinophils. Estimates from the weighted median estimator and MR-Egger did not differ substantially from those observed in IVW analyses. There was also no evidence of horizontal pleiotropy detected by the MR-Egger intercept (all *P* values >.10) (Figure 4).

Individual SNV associations with AD from EADB against the SNV exposure associations with types of leukocytes were visualized in scatterplots to provide the causal effect for each variant and the combined causal estimates of all variants from different methods (eFigure 4 in Supplement 1). Funnel plots showing the square root precision of each variant as a function of the causal effect estimate of the genetic variant showed an approximate symmetric distribution of the instrumental variables for each subpopulation of leukocytes (eFigure 5 in Supplement 1), suggesting no pleiotropy.

**Figure 5.**
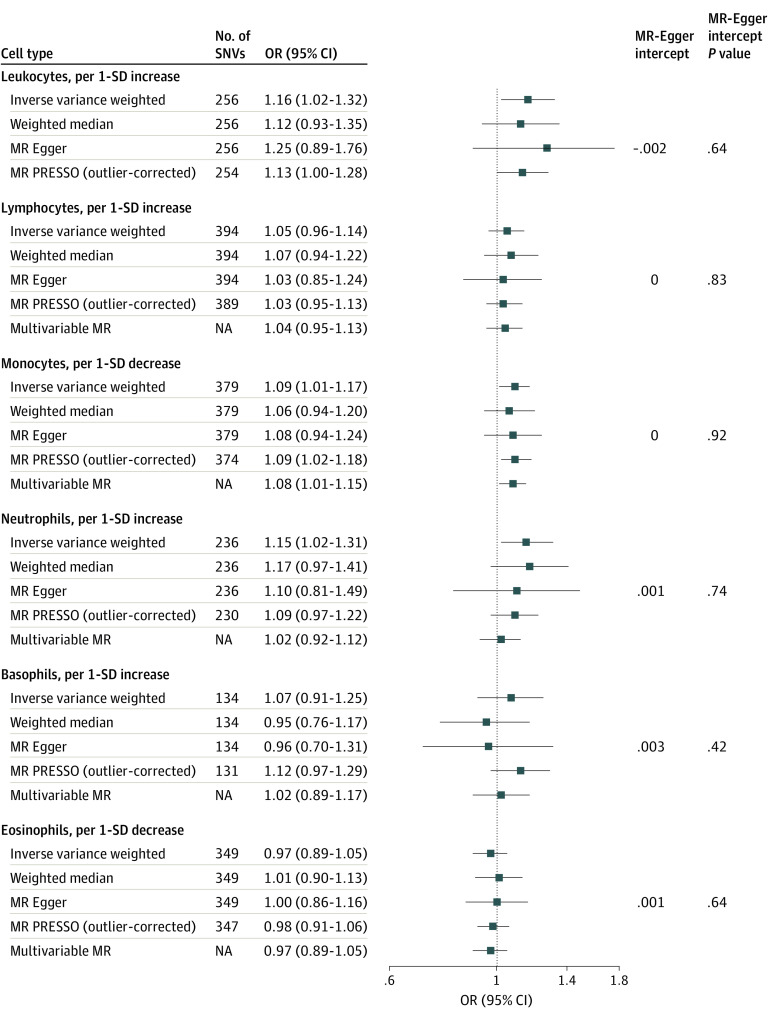
Mendelian Randomization (MR) Estimates of Types of Blood Leukocyte Counts on Risk of Alzheimer Disease Using the International Genomics of Alzheimer’s Project Estimated odds ratios (ORs) represent the effect of per 1-SD increase or decrease in different leukocyte counts on risk of Alzheimer disease obtained from different methods. *P* values for MR-Egger intercept represent horizontal pleiotropy. Total leukocyte count was not in the multivariable MR because the counts of subpopulations were derived from the total leukocyte count. NA indicates not applicable; SNVs, single nucleotide variants.

Results from the IGAP were generally similar to those obtained from the EADB, with ORs for AD of a 1-SD decrease of genetically determined blood monocyte counts 1.09 (95% CI, 1.01-1.17) in the IVW analysis, 1.09 (95% CI, 1.02-1.18) in the outlier-corrected MR PRESSO analysis, and 1.07 (95% CI, 1.01-1.15) in the MVMR analysis (Figure 5). In the IVW analysis, OR for AD for a 1-SD increase in leukocyte counts was 1.16 (95% CI, 1.02-1.32) and, for the neutrophil counts, 1.15 (95% CI, 1.02-1.31). However, after outlier removal in MR PRESSO analyses for blood leukocyte and neutrophil counts and in MVMR analyses for blood neutrophil counts, these associations were attenuated or no longer significant (Figure 5). Scatterplots of individual SNV effects and combined effects were visualized, and approximate symmetric funnel plots were obtained for each subpopulation of leukocyte counts (eFigure 6 and eFigure 7 in Supplement 1).

## Discussion

To our knowledge, no prospective study has examined the associations of types of blood leukocytes with incident AD. In the present study, observationally and genetically determined low blood monocyte counts were associated with a modestly increased risk of AD. The observational results were generated from a prospective cohort study of the Danish general population comprising 101 582 individuals, and the genetic results were generated by using individual-level data from the UK Biobank and the summary-level data from the largest GWAS of blood cell traits at the time of the study, including 563 085 individuals and 2 AD consortia totaling more than 1 million individuals. These findings are novel and suggest that low blood monocyte counts may reflect pathogenic pathways in AD.

Monocytes initiate Aβ clearance in the blood begins at approximately age 20 years, and this Aβ uptake ability may last throughout life, gradually decreasing with aging.^25^ At the prodromal or early stages of AD, monocyte activation exerts likely neuroprotective effects by promoting amyloid clearance in the blood, and thus reducing Aβ accumulation in the brain.^26^ This process probably continues for decades before clinical manifestation of AD. However, in advanced stages, the function of blood monocytes appears compromised with decreased phagocytosis capacity and shifts to a more proinflammatory state,^26^ and its overactivation by excessive Aβ aggregates will exacerbate neuroinflammation and promote AD progression. In patients with AD, monocytes showed poor macrophage differentiation ability and reduced Aβ phagocytosis,^27^ and monocyte receptors associated with Aβ internalization were decreased. For example, genetic variants associated with downregulated surface expression of triggering receptor expressed on myeloid cells, resulting in less binding of the triggering receptor expressed on myeloid cells ligands,^3^ and genetic variants associated with increased expression of CD33,^5^ both negatively affecting the phagocytic and Aβ clearance capacity, were associated with high risk of AD. This stage-dependent dual role of monocytes may explain the inconsistent results of blood monocyte counts in association with AD in studies with case-control or cross-sectional designs^14,15,16^ in which the associations were subject to various AD stages of the participants from different studies. A recent MR study did not find any association between types of leukocytes and AD^28^; however, this lack of association may be owed to insufficient statistical power.

The exact mechanism behind our observations is not completely clear. Nevertheless, substantial experimental findings support the notion that low blood monocyte counts may be important for AD pathogenesis. Patrolling monocytes in the blood were observed to target and clear Aβ by crawling onto the luminal walls of Aβ-positive veins, and selective removal of these monocytes resulted in Aβ accumulation in the brain.^9^ In addition, monocytes migrate into the brain where they differentiate into microglialike bone marrow–derived macrophages that are more efficacious than resident microglia in clearing Aβ.^29^ The recruitment of monocytes is primarily instigated through the C-C chemokine receptor type 2 (CCR2) pathway, where C-C motif chemokine ligand 2 (CCL2) secretion from activated microglia and astrocytes is triggered by Aβ.^30^ In mouse models, inhibition or depletion of CCR2 reduces the infiltration of monocytes into the brain, consequently increasing Aβ deposits.^31^ The expression of CCR2 in blood monocytes in patients with AD also decreased, whereas plasma CCL2 levels were increased and were associated with faster cognitive decline.^32^ Therefore, we suggest that a less efficient clearance of Aβ may consequently increase AD susceptibility, as reflected by a low blood monocyte count in the general population. Similarly, in our group’s previous work, both observationally and genetically determined low plasma levels of C-reactive protein were associated with high risk of AD.^33^ A mechanistic explanation could be that low C-reactive protein levels lead to decreased opsonization, decreased phagocytosis of Aβ by microglia, and decreased activation of the complement system, resulting in compromised Aβ elimination. We further looked into the mapped genes of the genetic instruments associated with monocyte counts in the MR analyses (eTable 7 in Supplement 2). The genes with the 10% highest weights in the current MR IVW analysis were associated with monocyte-macrophage function including migration, cytokines, and receptors, exemplified by, among others, *CCR2*, *EXOC3L2*, and *LYZ* (eTable 7 in Supplement 2). These genes have previously been reported to associate with immunodeficiency, AD, or amyloidosis, and thus further support the present findings.^31,34,35^ The present observed associations between peripheral markers of inflammation and the development of dementia may, however, not only directly be through AD pathology. Monocyte function may also predispose to other health conditions or comorbidities that ultimately influence cognition outside the AD pathogenesis.

### Strengths and Limitations

Strengths of our study include the large prospective general population cohort with no losses to follow-up, comprehensive information on potential confounding factors, and blood leukocyte counts at baseline preceding AD diagnosis. Furthermore, in genetic studies, we examined both individual-level data to test for nonlinearity and the largest summary-level data to generate the most robust estimates. The large sample sizes, particularly the AD case proportions, ensure a sufficient statistical power to detect even very small associations. In conventional MR, we selected instruments that were only associated with the examined blood leukocyte count to maximally avoid pleiotropy, and several sensitivity analyses accounting for invalid instruments, pleiotropy, and outliers were also conducted. In MVMR, we accounted for all types of leukocytes to address possible mediation by conditioning on all other types of leukocytes than the type of interest. The association remained for monocytes. The present observations for blood monocytes were also further strengthened by the observation that the genetic estimates became greater when using the exact AD diagnosis as in IGAP compared with the EADB consortium in which data on AD cases were merged with AD proxy cases from the UK Biobank.

The study has limitations. A potential limitation is the misclassification in the observational studies given that the individuals with AD were documented from hospital-based clinical diagnoses in the Danish registry. Nevertheless, AD diagnosis in Denmark is based on a combination of neurologic tests, cerebrospinal biomarkers, and imaging, together with clinical symptoms as recommended internationally.^36^ Furthermore, the validity of the AD diagnosis from the Danish Patient Registry has been demonstrated to be high.^37^ Results from the complete case analyses did not differ substantially from the main results, reassuring that the statistically significant differences observed between individuals with missing data vs those without missing data did not bias our results. In addition, compared with the associations of other well-established risk factors of AD, such as low educational attainment (OR, 0.64-0.89 for higher educational attainment),^38,39^ the estimate of low monocyte counts is modest. Finally, because the results were generated from White individuals of European ancestry, our findings may not apply to individuals of other races and ethnicities.

## Conclusions

The findings of this study suggest that low blood monocyte counts are associated with an increased risk of AD in the general population as noted both observationally and genetically. These findings highlight a potential role of the innate immune system in AD pathogenesis.

## Notes

Supplement 1. **eMethods.** Detailed Analysis **eTable 1.** Missing Value Percentage of Baseline Characteristics for Multiple Imputation **eTable 2.** Comparison of Participants With and Without Missing Data **eTable 3.** Baseline Characteristics of Participants in the Copenhagen General Population Study **eTable 4.** Total Leukocytes and Types of Leukocyte Counts by Percentile Groups and the Corresponding Absolute Levels of Cell Counts **eTable 5.** Summary Information of Genetic Instrumental Variables for Types of Leukocyte Counts From the Largest GWAS on Blood Cell Traits **eFigure 1.** Schematic Overview of the Study Design **eFigure 2.** Types of Blood Leukocyte Counts in Percentile Categories and Risk of Alzheimer Disease in Complete Case Analyses in the Copenhagen General Population Study (CGPS) **eFigure 3.** Power Calculation for MR Analyses **eFigure 4.** Scatterplots of MR Analyses From the European Alzheimer’s & Dementia Biobank **eFigure 5.** Funnel Plot of MR Analyses From the European Alzheimer’s & Dementia Biobank **eFigure 6.** Scatterplots of MR Analyses From the International Genomics of Alzheimer’s Project **eFigure 7.** Funnel Plot of MR Analyses From the International Genomics of Alzheimer’s Project
eReferences

Supplement 2.
Information of Instrumental Variables for Leukocyte Counts
**eTable 1.** Information of Instrumental Variables for Leukocyte Count **eTable 2.** Information of Instrumental Variables for Lymphocyte Count **eTable 3.** Information of Instrumental Variables for Monocyte Count **eTable 4.** Information of Instrumental Variables for Neutrophil Count **eTable 5.** Information of Instrumental Variables for Basophil Count **eTable 6.** Information of Instrumental Variables for Eosinophil Count **eTable 7.** Information of Mapped Genes of the Instrument Variables for Monocyte Count
