# Supplementary material for: Blood Leukocyte Counts in Alzheimer Disease
Source: JAMA Netw Open. 2022 Oct 10;5(10):e2235648. doi: 10.1001/jamanetworkopen.2022.35648 (PMC9552891; doi:10.1001/jamanetworkopen.2022.35648)
Supplement: Supplement 1. — eMethods. Detailed Analysis eTable 1. Missing Value Percentage of Baseline Characteristics for Multiple Imputation eTable 2. Comparison of Participants With and Without Missing Data eTable 3. Baseline Characteristics of Participants in the Copenhagen General Population Study eTable 4. Total Leukocytes and Types of Leukocyte Counts by Percentile Groups and the Corresponding Absolute Levels of Cell Counts eTable 5. Summary Information of Genetic Instrumental Variables for Types of Leukocyte Counts From the Largest GWAS on Blood Cell Traits eFigure 1. Schematic Overview of the Study Design eFigure 2. Types of Blood Leukocyte Counts in Percentile Categories and Risk of Alzheimer Disease in Complete Case Analyses in the Copenhagen General Population Study (CGPS) eFigure 3. Power Calculation for MR Analyses eFigure 4. Scatterplots of MR Analyses From the European Alzheimer’s & Dementia Biobank eFigure 5. Funnel Plot of MR Analyses From the European Alzheimer’s & Dementia Biobank eFigure 6. Scatterplots of MR Analyses From the International Genomics of Alzheimer’s Project eFigure 7. Funnel Plot of MR Analyses From the International Genomics of Alzheimer’s Project eReferences [file jamanetwopen-e2235648-s001.pdf]

Supplemental Online Content

Luo J, Thomassen JQ, Nordestgaard BG, Tybjærg-Hansen A, Frikke-Schmidt R.
Blood leukocyte counts in Alzheimer disease. *JAMA Netw Open.*
2022;5(10):e2235648. doi:10.1001/jamanetworkopen.2022.35648

eMethods. Detailed Analysis

eTable 1. Missing Value Percentage of Baseline Characteristics for Multiple Imputation

eTable 2. Comparison of Participants With and Without Missing Data

eTable 3. Baseline Characteristics of Participants in the Copenhagen General Population Study

eTable 4. Total Leukocytes and Types of Leukocyte Counts by Percentile Groups and the Corresponding Absolute Levels of Cell Counts

eTable 5. Summary Information of Genetic Instrumental Variables for Types of Leukocyte Counts From the Largest GWAS on Blood Cell Traits

eFigure 1. Schematic Overview of the Study Design

eFigure 2. Types of Blood Leukocyte Counts in Percentile Categories and Risk of Alzheimer Disease in Complete Case Analyses in the Copenhagen General Population Study (CGPS)

eFigure 3. Power Calculation for MR Analyses

eFigure 4. Scatterplots of MR Analyses From the European Alzheimer's & Dementia Biobank

eFigure 5. Funnel Plot of MR Analyses From the European Alzheimer's & Dementia Biobank

eFigure 6. Scatterplots of MR Analyses From the International Genomics of Alzheimer's Project

eFigure 7. Funnel Plot of MR Analyses From the International Genomics of Alzheimer's Project

eReferences

This supplemental material has been provided by the authors to give readers additional information about their work

© 2022 Luo J et al. *JAMA Network Open*.

eMethods

Observational analyses

Study population

The first recruitment of the Copenhagen General Population Study (CGPS) was initiated in 2003-2015. Follow-up examinations are ongoing. Briefly, White individuals aged 20 to 100+ at baseline were randomly selected from the national Danish Civil Registration System, where all Danish residents are recorded using a unique personal identification number. Thus, the participants are representative of the White Danish general population and due to the unique Danish registries, no individuals were lost to follow-up; those emigrating (n=453) were censored at the day of emigration. On the day of attendance, participants completed a self-administered questionnaire regarding information on lifestyle factors and medication use, a physical examination, and blood sampling for biochemical measurements, blood cell counts, and DNA extraction.

Blood leukocytes

All blood samples were analyzed on standard hospital clinical hematology equipment to estimate the cell counts. Regular internal and external quality control programs were performed. The samples were collected in standard vacutainers with EDTA as anticoagulant agent and measured using Advia systems (Siemens, Munich, Germany).

Alzheimer disease

Incident AD events were documented from the national Danish Patient Registry that provides data on all patient contacts with clinical hospital departments in Denmark since 1977, including outpatients and emergency wards since 1995. AD was defined according to the International Classification of Disease (ICD), comprising code 290.10 in ICD-8 and codes F00 and G30 in ICD-10. Follow-up time was from baseline to AD diagnosis, death, or censoring (emigration or the last update of the registries of 7th December 2018), whichever came first.

Covariates

The choice of covariates was primarily guided by empirical evidence (from previous studies) or theoretical knowledge of suspected or established confounding factors. The potential confounding factors were included in the Cox regression model one by one, and we compared the estimate changes of the exposure before and after introducing a certain confounding factor, as well as the models with and without this factor using a likelihood ratio test. If a potential confounding factor had notable influence, defined by a 10% change (well accepted in epidemiological studies) of the exposure estimates or a significant p value through model comparison, it was included in the final choice of adjusting covariates. Following this procedure, covariates from baseline include age, sex, body mass index (BMI, kg/m^2), apolipoprotein E (*APOE*) genotype, education, smoking, alcohol consumption, type 2 diabetes mellitus, and hypertension. Body mass index (BMI, kg/m^2) was computed as measured weight in kilograms divided by measured height in meters squared. Apolipoprotein E (*APOE*) was genotyped using Taqman-based assays and two loci were analyzed. The ε4 allele was p.Cys130Arg (rs429358) and the ε2 allele was p.Arg176Cys (rs7412). Subsequently, six common *APOE* genotype categories as ε22, ε32, ε42, ε33, ε43, and ε44 were generated. Education was categorized into three groups based on self-reported schooling years: <8 years, 8-12 years, and >12 years. Smoking status included self-reported current, former, and never smoker. Alcohol consumption was grouped into high (>14/21 units per week for women/men), moderate (3-14/21 units per week for women/men) and low (<3 units); one unit was equivalent to 12 grams of alcohol. Type 2 diabetes mellitus (yes/no) was defined as either self-reported disease, use of insulin or oral hypoglycemic agents, a non-fasting plasma glucose concentration of greater than 11 mmol/L, and/or a registry diagnosis at baseline. Hypertension (yes/no) was defined as self-reported use of blood pressure-lowering medication, high systolic blood pressure (≥140 mm Hg) or diastolic blood pressure (≥90 mm Hg), or a registry diagnosis at baseline.

Statistical analyses

All analyses were performed using R (v4.0.2) statistical software. Missing values (eTable 1 and eTable2 in the supplement) were imputed using multiple imputation by chain equations (MICE), setting the number of imputed datasets to 10. The imputation model included types of leukocyte counts, all covariates, the Nelson-Aalen estimator of cumulative hazard and incident AD status. Two multivariable-adjusted cause-

© 2022 Luo J et al. *JAMA Network Open*.

specific proportional hazards models were fitted within each imputed dataset and were subsequently pooled according to Rubin's rules to estimate hazard ratios (HR) and corresponding 95% confidence intervals (CI). The proportional hazard assumption was visually assessed by log(-log[survival]) versus log(analysis time) plots and was tested using Schoenfeld residuals. No major violations were observed. We fitted two multivariable-adjusted regression models, and in all models, age was used as the time scale (referred to as age adjustment in the following text), where subjects enter the analysis at their baseline age (left-truncation) and exit at their event/censoring/death age. Model 1 was adjusted for age and sex; model 2 was additionally adjusted for education, *APOE* genotype, BMI, smoking status, alcohol consumption, physical activity, hypertension, and type 2 diabetes mellitus. If only individuals with all data available (N = 98,318) were analyzed, results were similar to those reported.

Leukocyte counts were categorized into 5 groups by percentiles, i.e. <5th, $5-25^{th}$, $25-75^{th}$ (reference group), $75-95^{th}$, and >95th, to facilitate exploration of extreme leukocyte counts on risk of AD. The corresponding absolute leukocyte count for each cutoff point is shown in eTable 3 in the supplement. Moreover, on a linear scale, we generated restricted cubic splines (based on model 2) to illustrate the possible nonlinear dose-response relationship of different leukocyte counts with AD. Three knots located at the 5th, 50th, and 95th percentiles were selected in line with the categorical groups for an adequate fit of the model and to avoid overfitting.

Genetic analyses

Mendelian randomization

Mendelian randomization (MR) uses genetic variants associated with the exposure of interest to investigate the causal effect of risk factors on outcomes. Genetic variants inherited from parents to offspring at conception are randomly assorted and segregated at meiosis and are used as proxies of the exposure levels. As a result, individuals are divided into two comparable groups. Those who carry the effect allele (e.g. with an increased level of exposure) are assigned to the group with higher levels of the exposure of interest whereas those who carry the alternative allele are assigned to the group with lower levels of exposure. Hence, no confounding factors or reverse causation exists through the random allocation of genetic variants. Therefore, the difference in outcomes between genetically defined groups can be directly attributed to the exposure. Consequently, the MR design is considered as a "natural experiment", mimicking randomized clinical trials, where individuals are randomized to carry a genetic variant. To obtain reliable estimates of the effect of an exposure on an outcome from MR studies, genetic variants should meet three principal requirements, namely that they 1) are associated with the exposure; 2) are not related to any observed or unobserved confounding factors; 3) are associated with the outcome exclusively through its effect on exposure.

Genetic instruments for types of leukocyte cell counts

Leukocyte counts from the Blood Cell Consortium (BCX) was generated by impedance-based electronic cell counters; for lymphocytes, monocytes, neutrophils, basophils, and eosinophils, cell counts were the relative counts derived from the total leukocyte count multiplied by the proportion for each cell type. Genetic associations were performed using a linear mixed-effects model to account for relatedness in each cohort with the additive genetic model. Covariates included in the regression models were age, age-squared, sex, principal components, and study-specific factors such as study center. Single-nucleotide polymorphisms (SNPs) at a genome-wide significant level (p-value < 5×10^{-8}) were initially selected as potential genetic instrumental variables. To minimize possible pleiotropic bias, we excluded SNPs that were associated with more than one type of leukocyte cell count. In addition, linkage disequilibrium (LD) between SNPs for the same exposure was assessed in the European 1000 Genome Project reference panel. When LD was present ($r^2>0{\cdot}001$), the variant with the smallest P-value was retained. The total variation explained by the instrumental variables was calculated based on the retrieved summary statistics for each leukocyte count. We considered an F-statistic, calculated as $(beta/se)^2$, of above 10 to be a sufficiently strong instrument for reliable statistical analyses.

Non-linear MR

We calculated genetic risk scores (GRS) for each participant weighted by the associations of the genetic instrumental variables for different types of leukocyte counts identified from the previous step. For non-linear MR analyses, we first calculated instrument-free exposure, i.e., the residual variation of the continuous different types of leukocyte counts regressing on their GRS. Subsequently, we stratified on residual exposure to avoid overadjustment bias and collider bias[1]. We divided residual exposure into 20 equal groups by 5 percentiles (except for basophil and eosinophil due to the same value of some

© 2022 Luo J et al. *JAMA Network Open*.

percentiles), therefore, comparison could be made between individuals in the population who would have cell counts in the same stratum if they had the same genotype. GRS-AD associations were calculated within each stratum using logistic regression adjusted for age, sex, genotyping batch, and the first 10 principal components. GRS-cell counts associations are estimated using linear regression models among the whole population considering this association is constant, meaning that the association is similar across different strata. In the strata of the instrument-free exposure, the local average causal effect (LACE) of different types of leukocyte counts on AD is estimated by dividing GRS-AD associations by GRS-cell counts associations. We used the nlmr R package for the non-linear MR analyses (https://github.com/jrs95/nlmr)[2]. We adopted piecewise linear method in which a linear regression is fitted within each stratum of the instrument-free exposure. Confidence intervals are estimated through bootstrapping.

Data sources or Alzheimer disease

The European Alzheimer's & Dementia Biobank (EADB)

EADB brings together a range of European GWAS consortia, and summary estimates were based on 39,106 clinically diagnosed AD cases, 46,828 proxy-AD cases and 401,577 controls[3]. The diagnosis of cases varies from study to study but was generally diagnosed according to standard criteria including the Diagnostic and Statistical Manual of Mental Disorders (DSM III-R, IV) and the National Institute of Neurological and Communicative Disorders and Stroke and the Alzheimer's disease and Related Disorders Association (NINCDS-ADRDA) criteria, or based on the ICD-10 research criteria in registrations or health records. A more detailed description of diagnosis criteria of each participating study is available in the supplementary note of the GWAS[3]. Proxy-AD cases only presented in the UK Biobank, where proxy-AD designation was based on questionnaire data asking if parents had AD. Genetic associations were adjusted for age, sex, principal components and the number of *APOE* ε4 and ε2 alleles. The mean age of AD onset in cases among all included cohorts were between 63.2 and 83.7 years, and the mean age at examination of controls were between 44.9 to 82.8 years.

The International Genomics of Alzheimer's Project (IGAP)

To rule out the possible influence of proxy AD cases, we additionally used data from IGAP. IGAP meta-analyzed GWA studies on individuals of European ancestry comprising 21,982 cases and 41,944 cognitively normal controls from four consortia[4]. AD cases in individual studies were either confirmed through autopsies or based on a clinical diagnosis from health records in the participating studies. Genetic associations were adjusted for age, defined as age-at-onset for cases and age-at-last exam for controls, sex, and principal components. In the four cohorts, the mean age at onset of AD in cases ranged from 71.1 to 82.6 years, and the mean age at examination of controls ranged from 51.0 to 78.9 years.

Univariable Mendelian randomization analyses

We harmonized instruments-exposure and instruments-outcome data to ensure that effect estimates were aligned on the same effect allele, and palindromic genetic variants were eliminated. For the primary analysis, we used the inverse-variance weighted (IVW) method to combine SNP-specific estimates calculated by Wald ratios. IVW assumes no directional pleiotropic effect of each instrumental variable and constrains intercepts to zero. In addition, we performed several sensitivity analyses including weighted median estimator, MR-Egger regression, and Mendelian Randomization Pleiotropy RESidual Sum and Outlier (MR-PRESSO)[5-7]. Weighted median estimator can provide a reliable estimate if more than half of the instrumental variables are valid. In MR-Egger, the regression slope represents the causal effect of an exposure on an outcome, and the freely estimated intercept additionally provides an average magnitude of the pleiotropic effects across all genetic variants if it deviates from zero. MR-PRESSO was applied to detect and correct for horizontal pleiotropy through outlier removal in the instrumental variables, subsequently giving an unbiased causal estimate[7].

© 2022 Luo J et al. *JAMA Network Open*.

eTable 1. Missing value percentage of baseline characteristics for multiple imputation

Variables	Number of missing values	Missing percentage (%)
Leukocytes	514	0.47
Lymphocytes	535	0.49
Monocytes	537	0.50
Neutrophils	946	0.87
Basophils	518	0.48
Eosinophils	538	0.50
Body mass index	167	0.15
Education	363	0.34
Physical activity	861	0.80
Smoking status	777	0.72
Alcohol consumption	79	0.07
Hypertension	26	0.02
Type 2 diabetes mellitus	26	0.02

Variables with no missing values are not presented in the table.

© 2022 Luo J et al. *JAMA Network Open*.

eTable 2. Comparison of participants with and without missing data

	With missing data (N=3264)[1]	Complete cases (N=98,318)	P value[2]
Age (median [IQR]), years	60.7 (499, 70.4)	57.7 (47.9, 67.0)	<0.001
Sex (male, %)	1546 (47.4)	54,345 (55.3)	<0.001
Blood cell count (median [IQR]), 10^9/L			
Leukocytes	7.20 (6.10, 8.40)	7.00 (6.00, 8.20)	<0.001
Lymphocytes	2.10 (1.69, 2.57)	2.08 (1.71, 2.53)	0.5
Monocytes	0.40 (0.32, 0.50)	0.40 (0.33, 0.49)	0.4
Neutrophils	4.12 (3.39, 5.06)	4.03 (3.30, 4.93)	<0.001
Basophils	0.04 (0.02, 0.05)	0.04 (0.02, 0.05)	0.2
Eosinophils	0.17 (0.10, 0.26)	0.16 (0.11, 0.24)	0.3
Body mass index, (median [IQR]), kg/m^2	26.0 (23.6, 28.8)	25.5 (23.2, 28.4)	<0.001
APOE genotype, N (%)			0.7
ε22	24 (0.7)	687 (0.7)	
ε32	395 (12.1)	12,249 (12.5)	
ε33	1846 (56.6)	54,625 (55.6)	
ε42	101 (3.1)	2839 (2.9)	
ε43	799 (24.5)	25,074 (25.5)	
ε44	99 (3.0)	2844 (2.9)	
Education (years), N (%)			<0.001
< 8	474 (16.2)	9446 (9.6)	
8-12	1942 (66.6)	69,937 (71.1)	
> 12	501 (17.2)	18,935 (19.3)	
Physical activity (times per week), N (%)			0.8
≥ 4	1267 (51.8)	50,721 (51.6)	
< 4	1177 (48.2)	47,597 (48.4)	
Smoking status, N (%)			0.1
Current	474 (18.8)	16,972 (17.3)	
Ex-smoker	1007 (39.8)	39,513 (40.2)	
Non-smoker	1046 (41.4)	41,833 (42.5)	
Alcohol consumption (units per week), N (%)[3]			0.03
Light (< 3)	927 (29.1)	27,012 (27.5)	
Moderate (women: 3-14, men: 3-21)	1866 (58.5)	59,794 (60.8)	
Heavy (women: >14, men: >21)	397 (12.4)	11,512 (11.7)	
Hypertension, N (%)			<0.001
No	2257 (77.1)	78,961 (80.3)	
Yes	672 (22.9)	19,357 (19.7)	
Type 2 diabetes mellitus (%)			<0.001
No	3094 (94.9)	94,515 (96.1)	
Yes	167 (5.1)	3803 (3.9)	

Continuous variables are presented as median (interquartile range, IQR) and categorical variables are presented as frequency (percentage).
[1] Participants with missing values were defined as at least one missing value in any variable.

© 2022 Luo J et al. *JAMA Network Open*.

[2] P values were obtained from Wilcoxon rank-sum test for continuous variables, and chi-square test for categorical variables.

© 2022 Luo J et al. *JAMA Network Open*.

eTable 3. Baseline characteristics of participants in the Copenhagen General Population Study

	**N = 101,582**
Age (median [IQR]), years	57.8 (48.0, 67.1)
Male, %	45,691 (45.0)
Female (%)	55,891 (55.0)
Blood cell count (median [IQR]), 10^9/L	
Leukocytes	7.00 (6.00, 8.20)
Lymphocytes	2.08 (1.71, 2.53)
Monocytes	0.40 (0.33, 0.49)
Neutrophils	4.04 (3.30, 4.93)
Basophils	0.04 (0.02, 0.05)
Eosinophils	0.16 (0.11, 0.24)
Body mass index, (median [IQR]), kg/m^2	25.6 (23.2, 28.4)
APOE genotype, N (%)	
ε22	711 (0.7)
ε32	12,644 (12.4)
ε33	56,471 (55.6)
ε42	2940 (2.9)
ε43	25,873 (25.5)
ε44	2943 (2.9)
Education (years), N (%)	
< 8	9920 (9.8)
8-12	71,879 (70.8)
> 12	19,436 (19.1)
Physical activity (hours per week), N (%)	
≥ 4	51,988 (51.6)
< 4	48,774 (48.4)
Smoking status, N (%)	
Current	17,446 (17.3)
Former smoker	40,520 (40.2)
Non smoker	42,879 (42.5)
Alcohol consumption (units per week), N (%)	
Light (< 3)	27,939 (27.5)
Moderate (women: 3-14, men: 3-21)	61,660 (60.7)
Heavy (women: >14, men: >21)	11,909 (11.7)
Hypertension, N (%)	
No	40,661 (40.0)
Yes	60,897 (60.0)
Type 2 diabetes mellitus (%)	
No	97,609 (96.1)
Yes	3970 (3.9)

Continuous variables are presented as median (interquartile range, IQR) and categorical variables are presented as frequency (percentage).

© 2022 Luo J et al. *JAMA Network Open*.

eTable 4. Total leukocytes and types of leukocyte counts by percentile groups and the corresponding absolute levels of cell counts.

**Types of leukocyte**	**<5th percentile**	**5-25th percentile**	**25-75th percentile (reference)**	**75-95th percentile**	**>95th percentile**
Leukocytes (10^9/L)	1.0 - 4.8	4.8 - 6.0	6.1 - 8.2	8.3 - 10.4	10.5 - 97.5
Lymphocytes (10^9/L)	0.12 - 1.2	1.2 - 1.7	1.7 - 2.5	2.5 - 3.3	3.3 - 93.6
Monocytes (10^9/L)	0 - 0.24	0.25 - 0.33	0.33 - 0.49	0.49 - 0.66	0.67 - 6.7
Neutrophils (10^9/L)	0.04 - 2.4	2.4 - 3.3	3.3 - 4.9	4.9 - 6.7	6.7 - 53.6
Basophils (10^9/L)	0 - 0.01	0.01 - 0.02	0.02 - 0.05	0.05 - 0.08	0.09 - 2.03
Eosinophils (10^9/L)	0 - 0.06	0.07 - 0.11	0.12 - 0.24	0.25 - 0.43	0.43 - 13.6

© 2022 Luo J et al. *JAMA Network Open*.

eTable 5. Summary information of genetic instrumental variables for types of leukocyte counts from the largest GWAS on blood cell traits

Cell type	Determination	Unit	No. of Instruments	Explained Variation[1]	F statistics[2]
Leukocyte	Directly measured		256	3.4%	30 - 725
Lymphocyte	Percentage of each leukocyte type multiplied by the total leukocyte count	Per SD of log-10 transformed cell counts in nL	394	7.5%	30 - 2097
Monocyte			379	9.7%	30 - 2251
Neutrophil			236	3.4%	30 - 960
Basophil			134	2.6%	30 - 1717
Eosinophil			349	8.8%	30 - 1201

All SNPs included were independent (linkage disequilibrium $r^2<0.001$) and were associated with the relevant trait at a genome-wide significance level of $p<5\times10^{-8}$.
[1] Explained variation (R^2) was calculated by $2*MAF*(1-MAF)*\beta^2$, where β denotes the effect size and MAF denotes the minor allele frequency of the instrumental variable.
[2] Range of the F-statistics. GWAS: genome wide association study; MAF: minor allele frequency; SNPs: single-nucleotide polymorphism

© 2022 Luo J et al. *JAMA Network Open*.

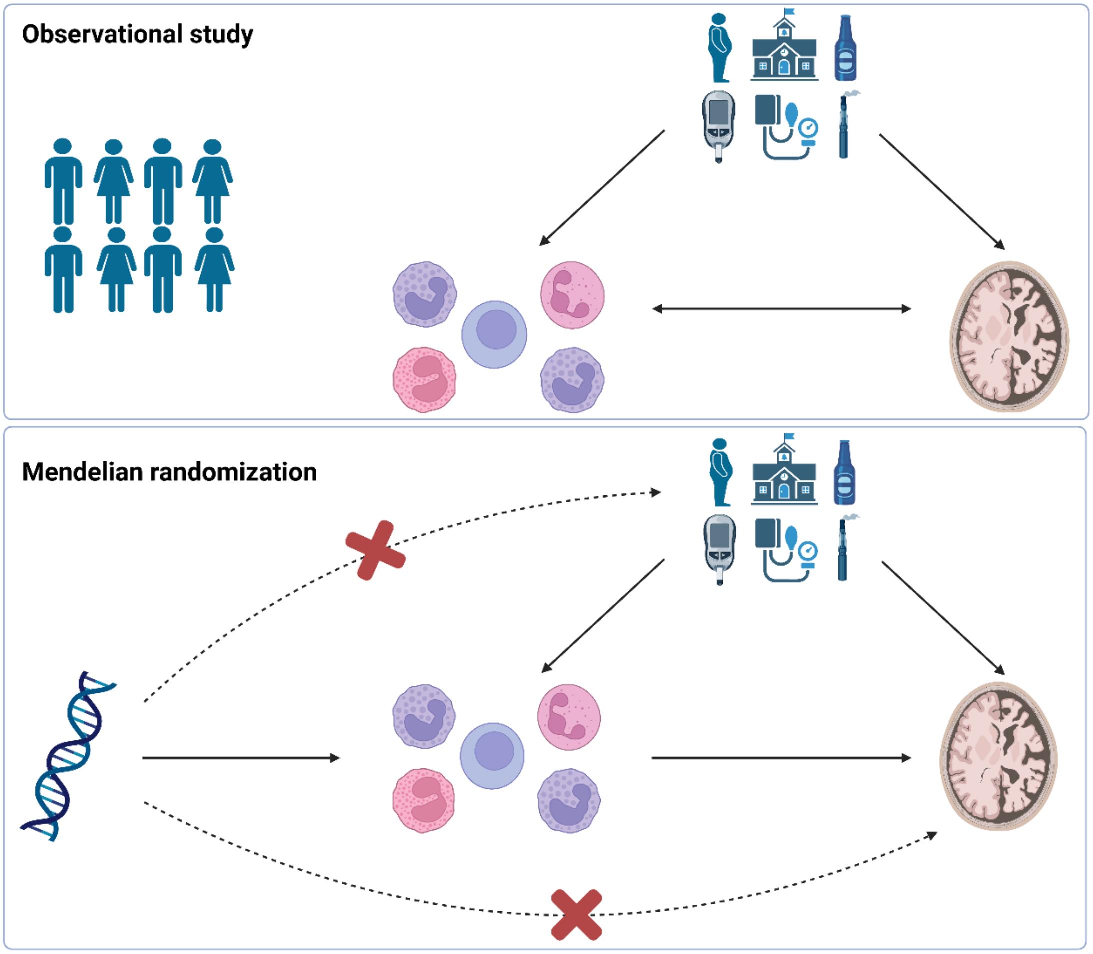

eFigure 1. Schematic overview of the study design

In this study, we used both observational (upper panel) and genetic studies (lower panel) to evaluate the associations between different types of leukocyte counts and risk of Alzheimer disease (AD). In observational studies, confounding factors that influence both the exposure and outcome may bias the true association between the exposure and the outcome; additionally, the observed association between the exposure and the outcome might be due to reverse causation, ordinarily referring to the situation in which the outcome precedes the exposure instead of the other way around. Therefore, the associations identified in observational studies cannot suggest causality. Genetic studies were performed by using Mendelian randomization (MR) approaches. The difference between MR and conventional observational studies is the use of genetic variants. Briefly, genetic variants associated with types of leukocyte counts at genome-wide significant level from the genome-wide association studies were exploited as instrumental variables for types of leukocyte counts. These genetic variants should not associate with any confounding factors and should associate with AD only through different types of leukocyte counts. Therefore, MR studies are free of confounding or reverse causation. The potential causal effect of different types of leukocyte counts on AD could be estimated by dividing gene-AD associations by gene-cell counts associations.

© 2022 Luo J et al. *JAMA Network Open*.

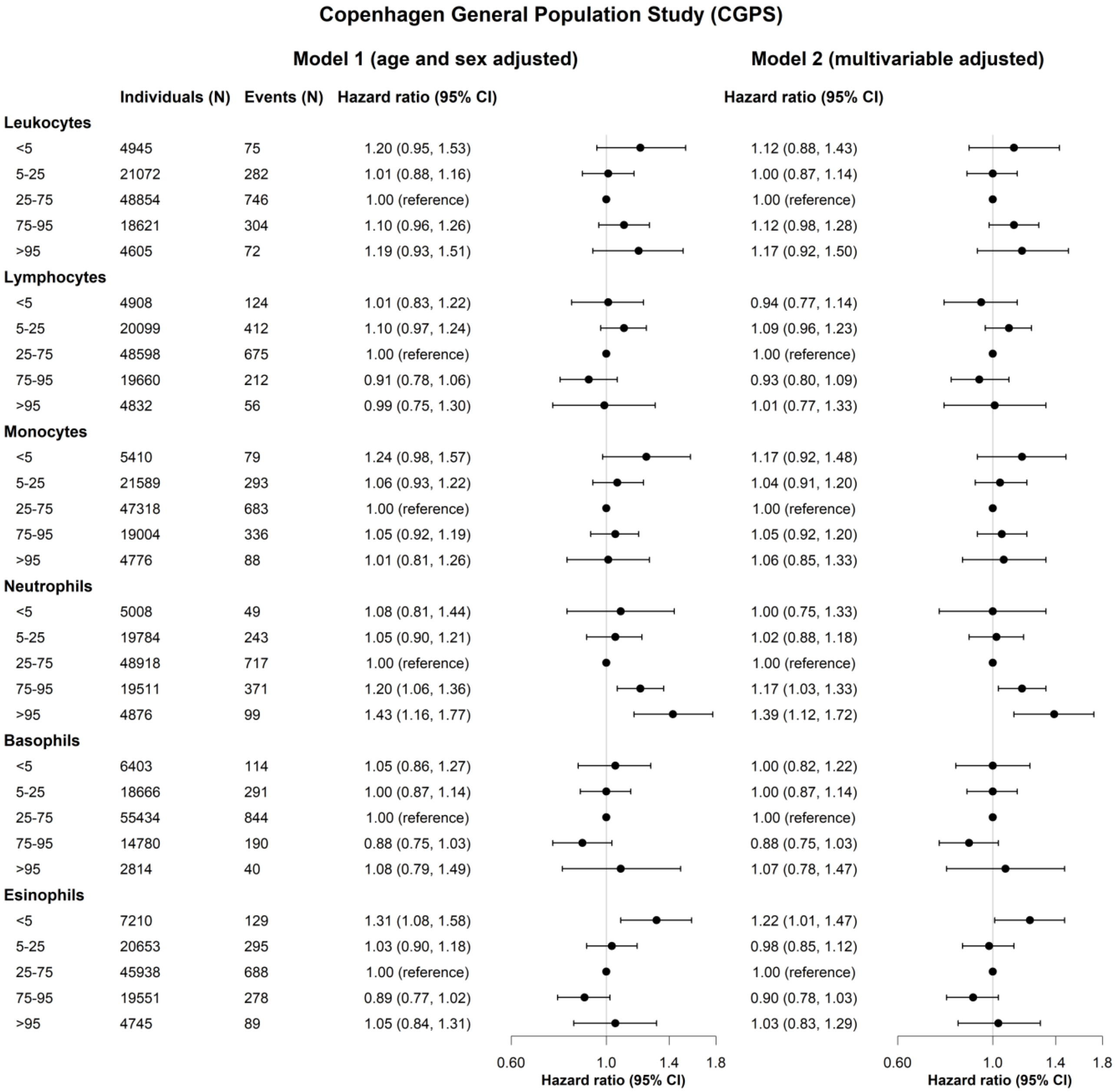

eFigure 2. Types of blood leukocyte counts in percentile categories and risk of Alzheimer disease in complete case analyses in the Copenhagen General Population Study

Cox regression analyses were performed among participants with no missing data (N=98,318). Cox regression model 1 was adjusted for age (time scale) and sex; model 2 was additionally adjusted for education levels, *APOE* ε2/3/4 genotype, and lifestyle factors including body mass index, smoking status, alcohol consumption, physical activity, hypertension and hypertensive medication, and type 2 diabetes mellitus. Hazard ratios for percentile groups were compared to the 25th-75th percentile group (reference). CI, 95% confidence interval.

© 2022 Luo J et al. *JAMA Network Open*.

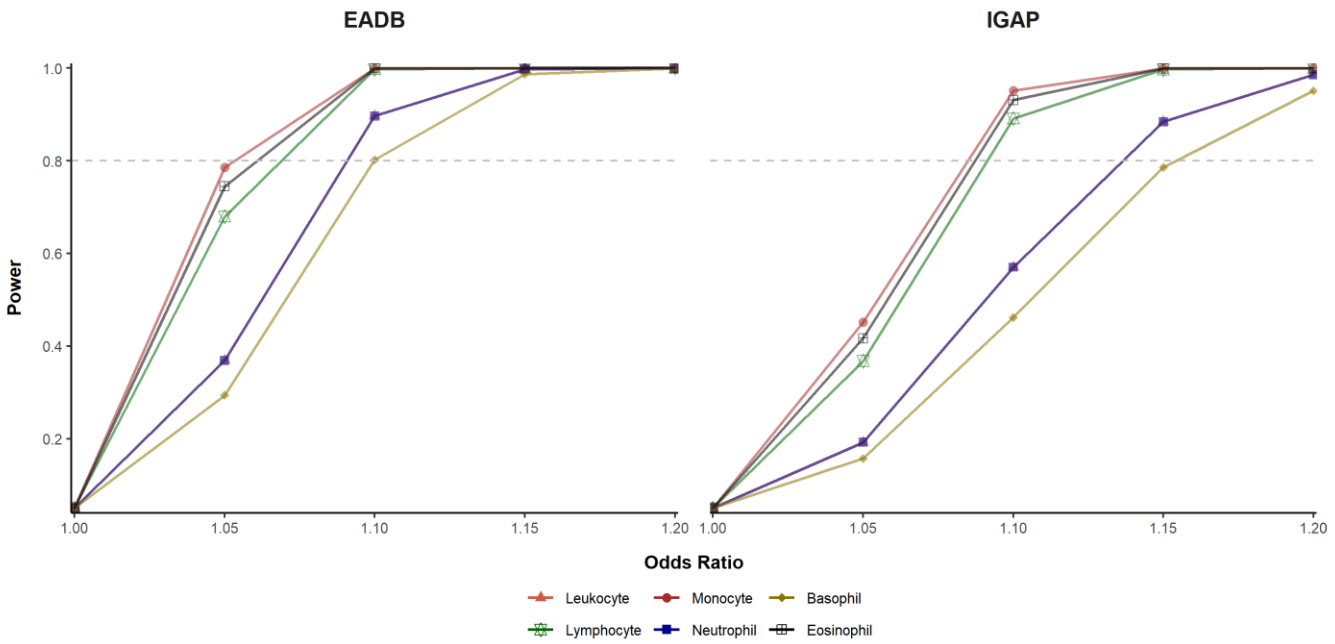

eFigure 3. Power calculation for Mendelian randomization analyses

Statistical power for MR analyses from the European Alzheimer's & Dementia Biobank (EADB) and the International Genomics of Alzheimer's Project (IGAP). Dashed grey line indicates statistical power of 80%. Variation explained by the instrumental variables for each cell count used for power calculation were listed in Table 2 using the online power calculation for MR (https://github.com/kn3in/mRnd).

© 2022 Luo J et al. *JAMA Network Open*.

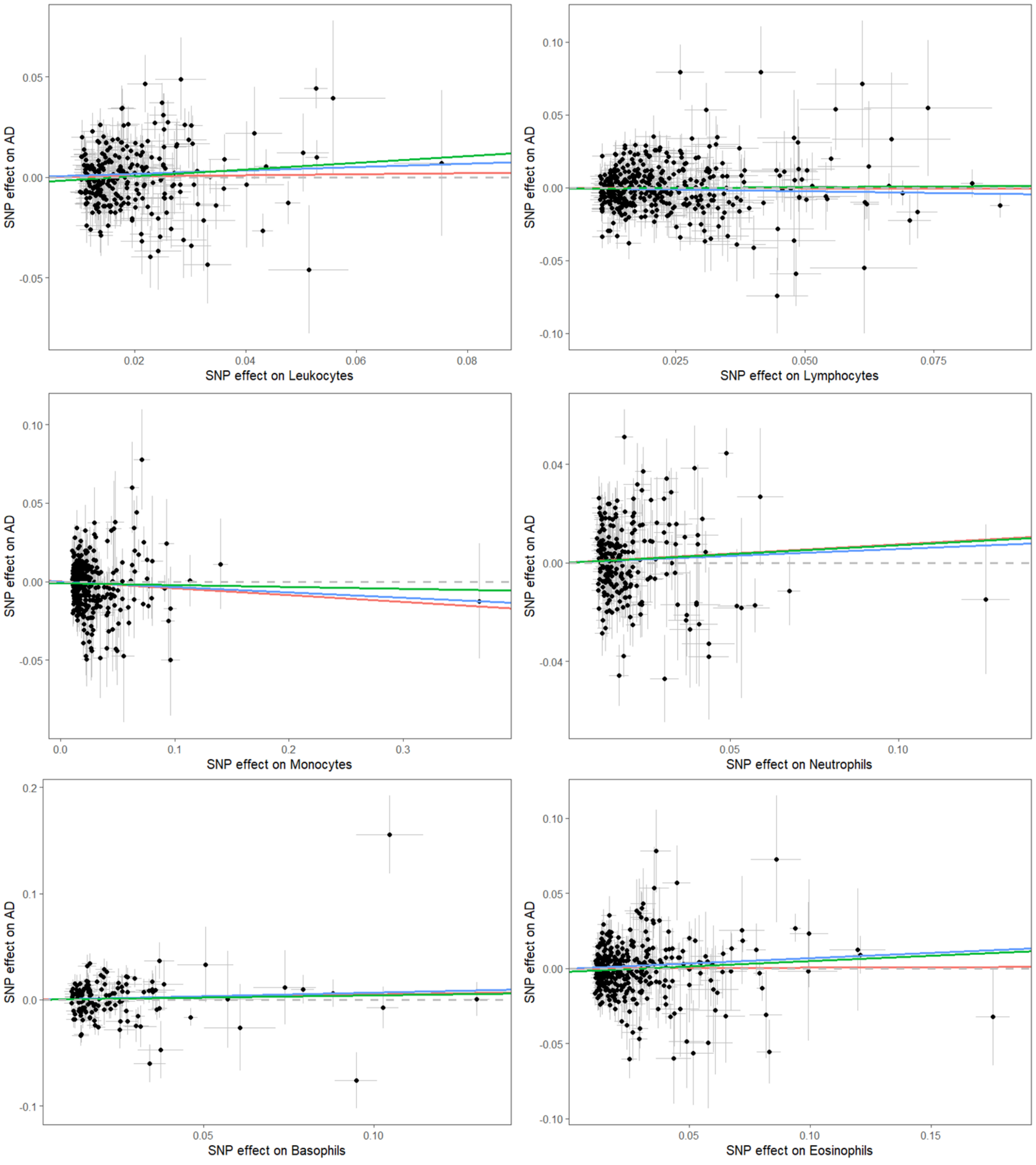

© 2022 Luo J et al. *JAMA Network Open*.

eFigure 4. Scatter plots of MR analyses from the European Alzheimer's & Dementia Biobank

The X-axis represents the SNP-exposure associations for each type of leukocytes while the Y-axis represents the SNP-AD associations from EADB. Black dots denote genetic instruments included in the primary MR analyses. Red: Inverse-variance weighted; Blue: Weighted-Median estimator; Green: MR-Egger.

© 2022 Luo J et al. *JAMA Network Open*.

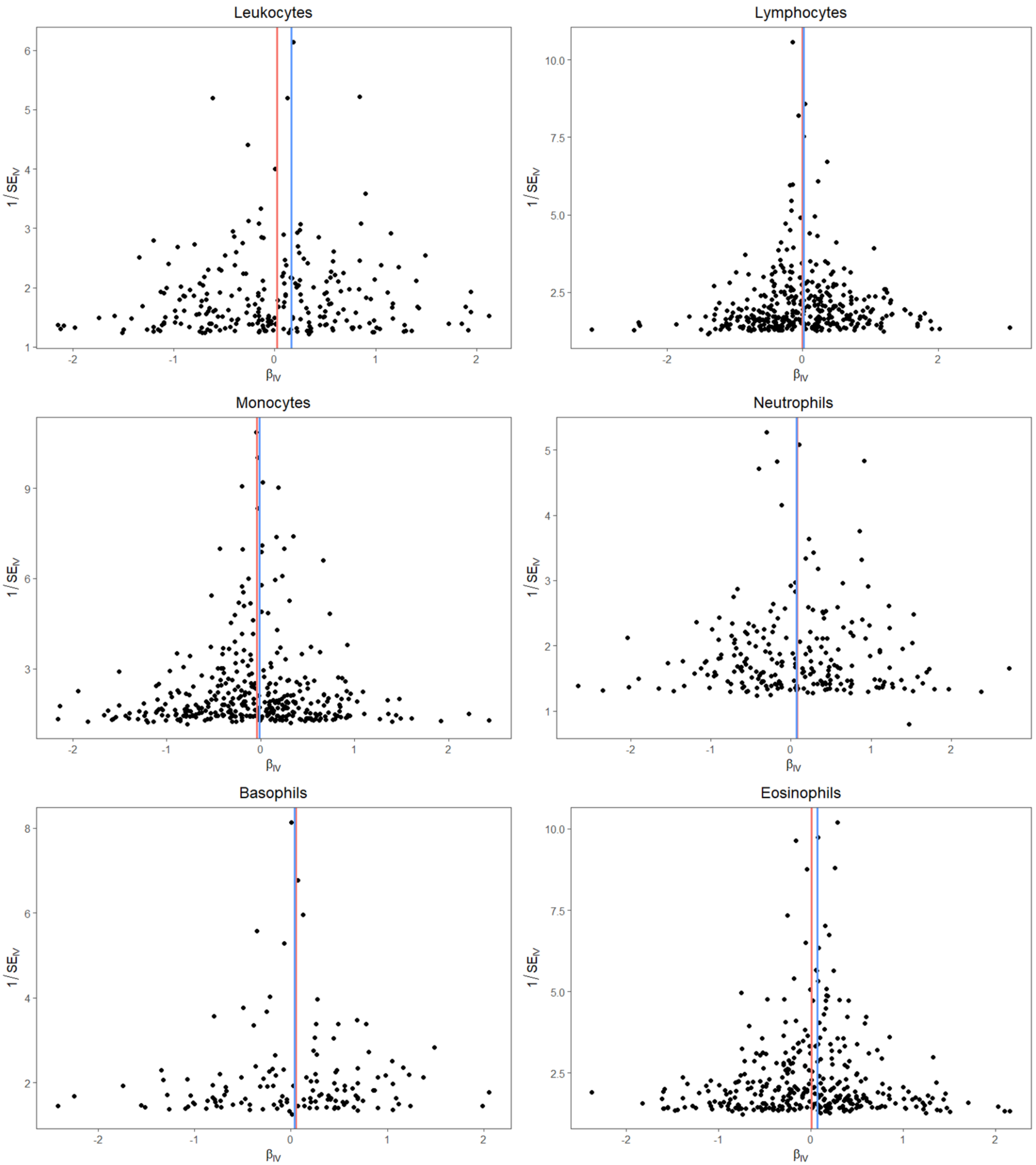

© 2022 Luo J et al. *JAMA Network Open*.

eFigure 5. Funnel plot of MR analyses from the European Alzheimer's & Dementia Biobank

Black dots denote genetic variants included in the primary MR analyses. The X-axis represents the causal effect estimate of the genetic variant and the Y-axis is the square root precision of each variant. Red: Inverse-variance weighted; Blue: MR-Egger.

© 2022 Luo J et al. *JAMA Network Open*.

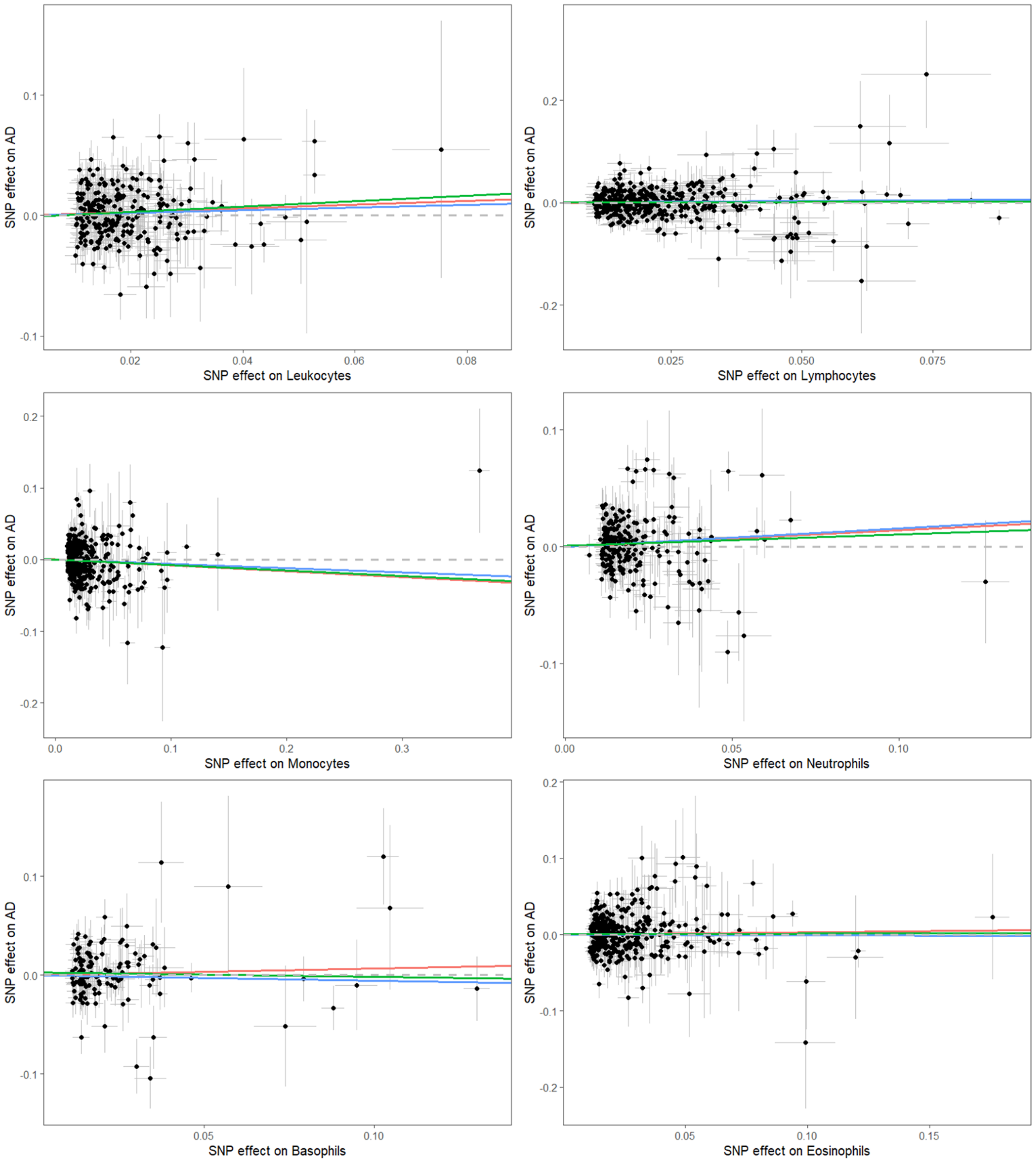

eFigure 6. Scatter plots of MR analyses from the International Genomics of Alzheimer's Project

The X-axis represents the SNP-exposure associations while the Y-axis represents the SNP-outcome associations. Black dots denote genetic instruments included in the primary MR analyses. Red: Inverse-variance weighted; Blue: Weighted-Median estimator; Green: MR-Egger.

© 2022 Luo J et al. *JAMA Network Open*.

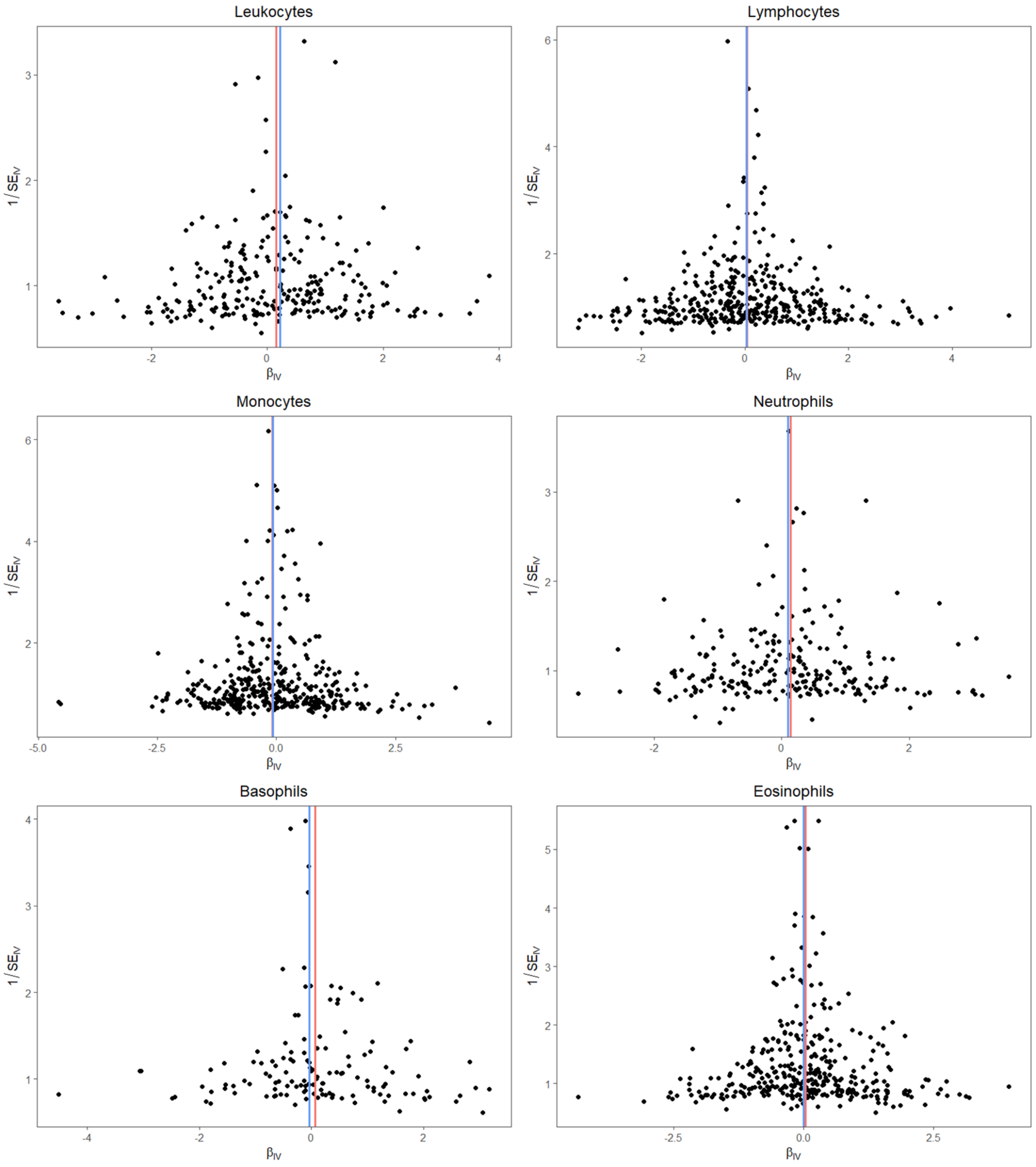

eFigure 7. Funnel plot of MR analyses from the International Genomics of Alzheimer's Project

Black dots denote genetic variants included in the primary MR analyses. The X-axis represents the causal effect estimate of the genetic variant and the Y-axis is the square root precision of each variant. Red: Inverse-variance weighted; Blue: MR-Egger.

© 2022 Luo J et al. *JAMA Network Open*.

eReferences

1. Sun YQ, Burgess S, Staley JR, Wood AM, Bell S, Kaptoge SK, et al. Body mass index and all cause mortality in HUNT and UK Biobank studies: linear and non-linear mendelian randomisation analyses. *Bmj* 2019; **364**: l1042.
2. Staley JR, Burgess S. Semiparametric methods for estimation of a nonlinear exposure-outcome relationship using instrumental variables with application to Mendelian randomization. *Genet Epidemiol* 2017; **41**(4): 341-52.
3. Bellenguez C, Küçükali F, Jansen IE, Kleineidam L, Moreno-Grau S, Amin N, et al. New insights into the genetic etiology of Alzheimer's disease and related dementias. *Nat Genet* 2022.
4. Kunkle BW, Grenier-Boley B, Sims R, Bis JC, Damotte V, Naj AC, et al. Genetic meta-analysis of diagnosed Alzheimer's disease identifies new risk loci and implicates Aβ, tau, immunity and lipid processing. *Nat Genet* 2019; **51**(3): 414-30.
5. Bowden J, Davey Smith G, Haycock PC, Burgess S. Consistent Estimation in Mendelian Randomization with Some Invalid Instruments Using a Weighted Median Estimator. *Genet Epidemiol* 2016; **40**(4): 304-14.
6. Bowden J, Davey Smith G, Burgess S. Mendelian randomization with invalid instruments: effect estimation and bias detection through Egger regression. *Int J Epidemiol* 2015; **44**(2): 512-25.
7. Verbanck M, Chen CY, Neale B, Do R. Detection of widespread horizontal pleiotropy in causal relationships inferred from Mendelian randomization between complex traits and diseases. *Nat Genet* 2018; **50**(5): 693-8.

© 2022 Luo J et al. *JAMA Network Open*.
